# Epigenetic clock and methylation studies in vervet monkeys

**DOI:** 10.1007/s11357-021-00466-3

**Published:** 2021-09-30

**Authors:** Anna J. Jasinska, Amin Haghani, Joseph A. Zoller, Caesar Z. Li, Adriana Arneson, Jason Ernst, Kylie Kavanagh, Matthew J. Jorgensen, Julie A. Mattison, Kevin Wojta, Oi-Wa Choi, Joseph DeYoung, Xinmin Li, Andrew W. Rao, Giovanni Coppola, Nelson B. Freimer, Roger P. Woods, Steve Horvath

**Affiliations:** 1 Center for Neurobehavioral Genetics, Semel Institute for Neuroscience and Human Behavior, Department of Psychiatry and Biobehavioral Sciences, David Geffen School of Medicine, University of California, Los Angeles, Los Angeles, CA USA; 2 Institute of Bioorganic Chemistry, Polish Academy of Sciences, Poznan, Poland; 3 Eye On Primates, Los Angeles, CA USA; 4 Department of Human Genetics, David Geffen School of Medicine, University of California, Los Angeles, Los Angeles, CA USA; 5 Department of Biostatistics, School of Public Health, University of California, Los Angeles, Los Angeles, CA USA; 6 Interdepartmental Bioinformatics Program, University of California, Los Angeles, Los Angeles, CA 90095 USA; 7 Department of Biological Chemistry, University of California, Los Angeles, Los Angeles, CA 90095 USA; 8 Department of Pathology, Section On Comparative Medicine, Wake Forest School of Medicine, Medical Center Boulevard, Winston-Salem, 27157-1040 USA; 9 Department of Biomedical Sciences, University of Tasmania, Hobart, Australia; 10 Translational Gerontology Branch, National Institute On Aging Intramural Research Program, National Institute of Health, Gaithersburg, USA; 11 Department of Neurology, David Geffen School of Medicine, UCLA, Los Angeles, USA; 12 Technology Center for Genomics & Bioinformatics, Department of Pathology & Laboratory Medicine, University of California, Los Angeles, Los Angeles, CA 90095 USA

**Keywords:** Vervet, Monkey, Aging, Development, Epigenetic clock, DNA methylation

## Abstract

DNA methylation-based biomarkers of aging have been developed for many mammals but not yet for the vervet monkey (*Chlorocebus sabaeus*), which is a valuable non-human primate model for biomedical studies. We generated novel DNA methylation data from vervet cerebral cortex, blood, and liver using highly conserved mammalian CpGs represented on a custom array (HorvathMammalMethylChip40). We present six DNA methylation-based estimators of age: vervet multi-tissue epigenetic clock and tissue-specific clocks for brain cortex, blood, and liver. In addition, we developed two dual species clocks (human-vervet clocks) for measuring chronological age and relative age, respectively. Relative age was defined as ratio of chronological age to maximum lifespan to address the species differences in maximum lifespan. The high accuracy of the human-vervet clocks demonstrates that epigenetic aging processes are evolutionary conserved in primates. When applying these vervet clocks to tissue samples from another primate species, rhesus macaque, we observed high age correlations but strong offsets. We characterized CpGs that correlate significantly with age in the vervet. CpG probes that gain methylation with age across tissues were located near the targets of Polycomb proteins SUZ12 and EED and genes possessing the trimethylated H3K27 mark in their promoters. The epigenetic clocks are expected to be useful for anti-aging studies in vervets.

**Supplementary Information:**

The online version contains supplementary material available at 10.1007/s11357-021-00466-3.

## Introduction

Non-human primates (NHPs) are regarded as critical animal models used in biomedical research [1–6] and key reference species used for constructing a comparative framework essential for evolutionary biology studies [7, 8]. NHPs, as compared with rodents, more closely resemble humans in terms of lifespan, life history strategies, cognitive processes, immunological behaviors [9], inflammatory responses [10], and other health characteristics that are relevant to aging processes [11]. Therefore, NHPs are invaluable models for studying the pathomechanisms of age-related diseases, developing novel anti-aging treatments, and performing preclinical testing of such therapies before translation to human subjects [12]. Therefore, specialized tools are needed for the assessment of aging processes in NHP models in the context of the environmental and genetic factors regulating natural aging, the pathogenesis of age-related conditions, and the development and testing of anti-aging therapies.

On the molecular level, the process of aging is associated with epigenetic DNA modifications, such as DNA methylation (DNAm) of cytosine residues within CpG dinucleotides (5-methyl-cytosine) across the genome.

DNA methylation levels have been used to develop multi-tissue estimators of chronological age and mortality risk [13–18]. Whereas physiological conditions (e.g., BMI and menopause), pathologies (e.g., cancers and neurodegenerative diseases), and environmental factors (e.g., diet, exercise, and HIV infection) can affect the trajectory of DNAm age [15, 19–23], the pace of DNAm age is a heritable genetic trait linked to several genomic regions [24–26].

Epigenetic clocks have been developed for several NHPs including rhesus macaque and marmoset [27, 28] but not yet for the vervet monkey. The vervet monkey (genus *Chlorocebus*) is an Old World monkey frequently used as a model in biomedical research [1, 6] particularly for complex chronic diseases, many of which either are associated with aging or aggravate the process of aging. Vervets exhibit some aspects of human aging, including neurodegeneration [29–32], and reproductive senescence and menopause [33]. Vervets have also been used in studies of reproductive physiology and obesity [34], the effects of genes and diet on growth and obesity [35], cardiometabolic health [36], physiological and behavioral stress responses [37–39], and multi-tissue genetic regulation of gene expression, including that in tissues involved in stress responses [40]. Because it is a natural host of simian immunodeficiency virus, which typically does not progress to immunodeficiency upon infection, the vervet is an established model for AIDS research [41–44]. Whereas HIV infection in humans is associated with age acceleration [19, 45], the links between the benign course of simian immunodeficiency virus infection and aging in the vervet remain unknown.

Vervets from diverse African populations and the bottlenecked founder populations in the Caribbean have been phylogenetically characterized and, together with the genetically characterized extended pedigree of Caribbean-origin vervets in the Vervet Research Colony (VRC) at Wake Forest School of Medicine, are used for genetic, gene-phenotype, developmental, and infectious disease studies [40, 46–53]. To advance use of the vervet as a model for developmental and aging studies and facilitate DNAm-based assessments of the age effects of various environmental exposures, including preclinical testing of anti-aging therapies, here we created a multi-tissue epigenetic age estimator for the vervet, which is based on the blood, liver, and brain prefrontal cortex. Given that chronological ages are difficult to assess in free ranging monkeys, the epigenetic clock can also enable objective age assessment in wild vervet populations.

## Methods

### Study subjects

All animals used in this study were Caribbean-origin vervet monkeys (*Chlorocebus sabaeus*) from the VRC at Wake Forest School of Medicine. The VRC colony is an extended multigenerational pedigree established from 57 founders imported from the islands of St. Kitts and Nevis in the West Indies. The introduction of new animals to the pedigree ended in the mid-1980s [1]. The colony members are socially reared in extended family groups mimicking the natural social composition of vervet monkey troops in the wild. Group sizes range from 11 to 23 animals, with one or two intact adult males included in each group. Unfamiliar males are rotated into each group every 3–5 years. The pedigree structure is genetically confirmed [48]. All colony-born vervets have known chronological age accurate to 1 day.

Beyond applications in aging studies, animals from VRC are used in a wide range of research in areas such as the efficacy and enhancement of vaccines for infectious diseases, e.g., influenza and dengue [54–56]; investigations of diabetes, metabolic disease, and obesity [57–59]; and the development of novel non-invasive biomedical imaging methodologies [60, 61].

### Ethics statement

The Wake Forest School of Medicine facilities are certified by the Association for Assessment and Accreditation of Laboratory Animal Care. The animal handling and sample collection procedures in this study were performed by a veterinarian after review and approval by the UCLA and VA Institutional Animal Care and Use Committees. Both housing and sample collection were in compliance with the US National Research Council Committee’s Guidelines for Care and Use of Laboratory Animals [62] and met or exceeded all standards of the Public Health Service’s “Policy on the Humane Care and Use of Laboratory Animals” [63].

### Vervet tissue samples

For this study, we selected a total of 240 samples representing the entire vervet lifespan, from neonatal to senile stages: 144 samples from the peripheral blood, 48 samples from the liver, and 48 samples from the cortical brain area BA10. The brains were perfused to remove blood prior to dissection [40]. The targeted brain area BA10 was very small, and brain samples were dissected as bulk tissues, collecting, to the extent feasible without the benefit of microscopy, the full thickness of the cortex while avoiding the underlying white matter [40]. One outlier blood sample (202943350003_R03C01 from animal 1,992,020) was excluded from analysis on the basis of the DNAm profile. The remaining 143 blood samples included 14 pairs of biological replicates collected from 14 individuals at two different time points 3.9–10.93 years apart. Peripheral blood was collected through venipuncture with standard procedures. Liver and brain cortical tissues were collected during necropsies [40].

Genomic DNA was isolated from blood and liver samples primarily through Puregene chemistry (Qiagen). DNA from the liver was extracted manually and that from the blood was extracted with an automated Autopure LS system (Qiagen). DNA was extracted from old liver tissues and clotted blood samples manually with a QIAamp DNA Blood Midi Kit and DNeasy Tissue Kit according to the manufacturer’s protocol (Qiagen, Valencia, CA). DNA from BA10 was extracted on an automated nucleic acid extraction platform AnaPrep (Biochain) with a magnetic bead-based extraction method and Tissue DNA Extraction Kit (AnaPrep).

### Human tissue samples

To build the human-rhesus macaque clock, we analyzed previously generated methylation data from *n* = 1207 human tissue samples (adipose, blood, bone marrow, dermis, epidermis, heart, keratinocytes, fibroblasts, kidney, liver, lung, lymph node, muscle, pituitary, skin, spleen) from individuals whose ages ranged from 0 to 93 years. These human methylation data are described in [64]. The tissue samples came from three sources. Tissue and organ samples were from the National NeuroAIDS Tissue Consortium [65]. Blood samples were from the Cape Town Adolescent Antiretroviral Cohort study [45]. Skin and other primary cells were provided by Kenneth Raj [66]. Ethics approval (IRB#15–001,454, IRB#16–000,471, IRB#18–000,315, IRB#16–002,028).

### Rhesus tissue samples

To validate the vervet clock cross species, we utilized the CpG methylation data described in a companion paper [28].

### DNA methylation data

All DNA methylation data were generated using the custom Infinium array “HorvathMammalMethylChip40” [67]. By design, the mammalian methylation array facilitates epigenetic studies across mammalian species (including rhesus macaques and humans) due to its very high coverage (over thousandfold) of highly conserved CpGs in mammals. In addition, the custom array contains 1951 CpGs selected from human biomarker studies. The particular subset of species for each probe is provided in the chip manifest file that can be found at Gene Expression Omnibus (GEO) at NCBI as platform GPL28271. Not all of the CpGs on the array apply to vervet monkeys. Only 36,727 CpGs out of all CpGs on the mammalian array actually map to the vervet genome according to the ChlSab1.1.100 genome assembly from ENSEMBL. The SeSaMe normalization method was used to define beta values for each probe [68].

### Penalized regression models

Details on the clocks (CpGs, genome coordinates) and R software code are provided in the Supplement.

Penalized regression models were created with glmnet [69]. We investigated models produced by “elastic net” regression (alpha = 0.5). The optimal penalty parameters in all cases were determined automatically by using a tenfold internal cross-validation (cv.glmnet) on the training set. By definition, the alpha value for the elastic net regression was set to 0.5 (midpoint between Ridge and Lasso type regression) and was not optimized for model performance.

We performed a cross-validation scheme for arriving at unbiased (or at least less biased) estimates of the accuracy of the different DNAm-based age estimators. For validation of the clocks, we used leave-one-out cross-validation (LOOCV) in which one sample was left out of the regression, then predicted the age for the remaining samples, and iterated this process over all samples.

A critical step is the transformation of chronological age (the dependent variable). While no transformation was used for the multi-tissue clock for vervets, we did use a log linear transformation for the dual species clock of chronological age (Supplement).

### Relative age estimation

To introduce biological meaning into age estimates of vervets and humans that have very different lifespan, as well as to overcome the inevitable skewing due to unequal distribution of data points from vervets and humans across age range, relative age estimation was made using the formula: Relative age = Age/maxLifespan where the maximum lifespan for the two species was chosen from the *anAge* database [70]. Maximum age of vervets and humans was 30.8 and 122.5 years, respectively.

### Epigenome-wide association studies of age

EWAS was performed in each tissue separately with the R function “standardScreeningNumericTrait” in the “WGCNA” R package [71]. Next, the results were combined across tissues with Stouffer’s meta-analysis method.

### CpG set enrichment analysis

The significant CpGs for each tissue were selected for enrichment analysis. The first enrichment analysis was done for transcriptional factor motifs. Using the MEME motif discovery algorithm [72], we predicted the probes that are located on TF motifs from five databases: Jasper, Taipale, Taipaledimer, Uniprob, and TRANSFAC. The overlap of selected CpGs based on the EWAS was tested with the predicted background using a hypergeometric test.

### Genome annotation

The gene-level enrichment was done using GREAT analysis [73] and human Hg19 background. The background probes were limited to 24,799 probes that were mapped to the same gene in the Vervet Monkey genome. Gene set enrichment was done for gene ontology, molecular pathways, diseases, upstream regulators, and human and mouse phenotypes.

We aligned microarray probes to the vervet reference genome Chlorocebus_sabeus 1.1 GCF_000409795.2 [47]. CpG sites were annotated in relation to the nearest genes based on the vervet gene annotations: Ensembl *Chlorocebus sabaeus* Annotation Release 100 [74]. In total, 35,898 probes from the mammalian BeadChip array could be aligned to ChlSab1.1.100 genome (Ensembl). The alignment was done using the QUASR package [75], with the assumption for bisulfite conversion treatment of the genomic DNA. Following the alignment, the CpGs were annotated based on the distance to the closest transcriptional start site using the Chipseeker package [76].

## Results

To identify age-related CpGs and develop a multi-tissue epigenetic age predictor for vervet monkeys, we leveraged developmental tissue resources from the VRC vervets comprising animals representing the entire vervet lifespan, from neonates to senile individuals, with known chronological ages accurate to 1 day as detailed in Table 1. We characterized DNAm in three tissues: the peripheral blood (*N* = 240, from 1 day to 25 years of age), a classical immune tissue that is available through minimally invasive sampling and is routinely used for biomarker studies [46, 77]; the liver (*N* = 48, from 0 day to 21 years of age), a key metabolic organ; and a region of the prefrontal cortex in the brain corresponding to the Brodmann area 10 (*N* = 48, from 0 day to 22 years of age), a subregion implicated in personality expression and executive function. We generated high-quality DNAm profiles from these samples using 36,727 CpGs located at highly conserved regions in the mammals represented on the HorvathMammalMethylChip40 [67].

**Table 1:** Description of the data by tissue type

Tissue	*N*	No. female	Mean age	Min. age	Max. age
Blood	144	100	10.2	0.0027	25
Cortex	48	25	3.15	0	22.9
Liver	48	28	2.81	0	21.8

*N* total number of tissues. Number of females. Age: mean, minimum, and maximum

### Samples cluster by tissue type

Unsupervised hierarchical clustering of tissue samples on the basis of all tested CpG sites revealed three distinct clusters, one for each tissue type (Supplementary Fig. 1). The clusters from the peripheral tissues, blood, and liver were grouped together, whereas the brain cortex cluster was more distant. Within the liver cluster, the samples from animals older than 8.7 years (*N* = 7 individuals) formed a separate subcluster, thus suggesting marked differences in DNAm profiles between fully adult individuals versus immature individuals and young adults in this organ. The observations in the vervets supported previous results in humans showing that extensive tissue-specific remodeling of DNAm patterns occurs in the liver during aging [78].

### Epigenetic clocks

We used these high-quality DNAm data to construct different epigenetic clocks for vervet only and for both human and vervet. For the construction of the dual human-vervet clock, we used the DNAm data previously generated with the HorvathMammalMethylChip40 in 1211 human samples representing 16 tissues from individuals 0 to 93 years old [45, 65, 66]. Our clocks for vervet monkeys can be distinguished along three dimensions (tissue type, species, and measure of age). We used a combined set of all samples to train a multi-tissue clock (pan-clock) suited for age predictions across different tissue types included in the clock construction. We also created clocks tailor-made for specific tissues/organs, which were trained on the basis of the samples from individual tissue types: the blood-clock, the liver-clock, and the brain cortex-clock. We anticipate that pan-clock may provide a proxy for tissues for which tissue-specific clocks are not available.

While the multi-tissue vervet clock applies only to vervets, we also created dual species clocks, referred to as human-vervet clocks, for estimates of chronological age and relative age. Relative age is the ratio of chronological age to maximum lifespan (i.e., the maximum age of death observed in the species). Thus, relative age takes on values between 0 and 1. The maximum lifespan observed for humans and vervets was 122.5 and 30.8 years, respectively. Relative age allows alignment and biologically meaningful comparison between species with different lifespan (vervet and human), which is not afforded by mere measurement of chronological age.

**Fig. 1:**
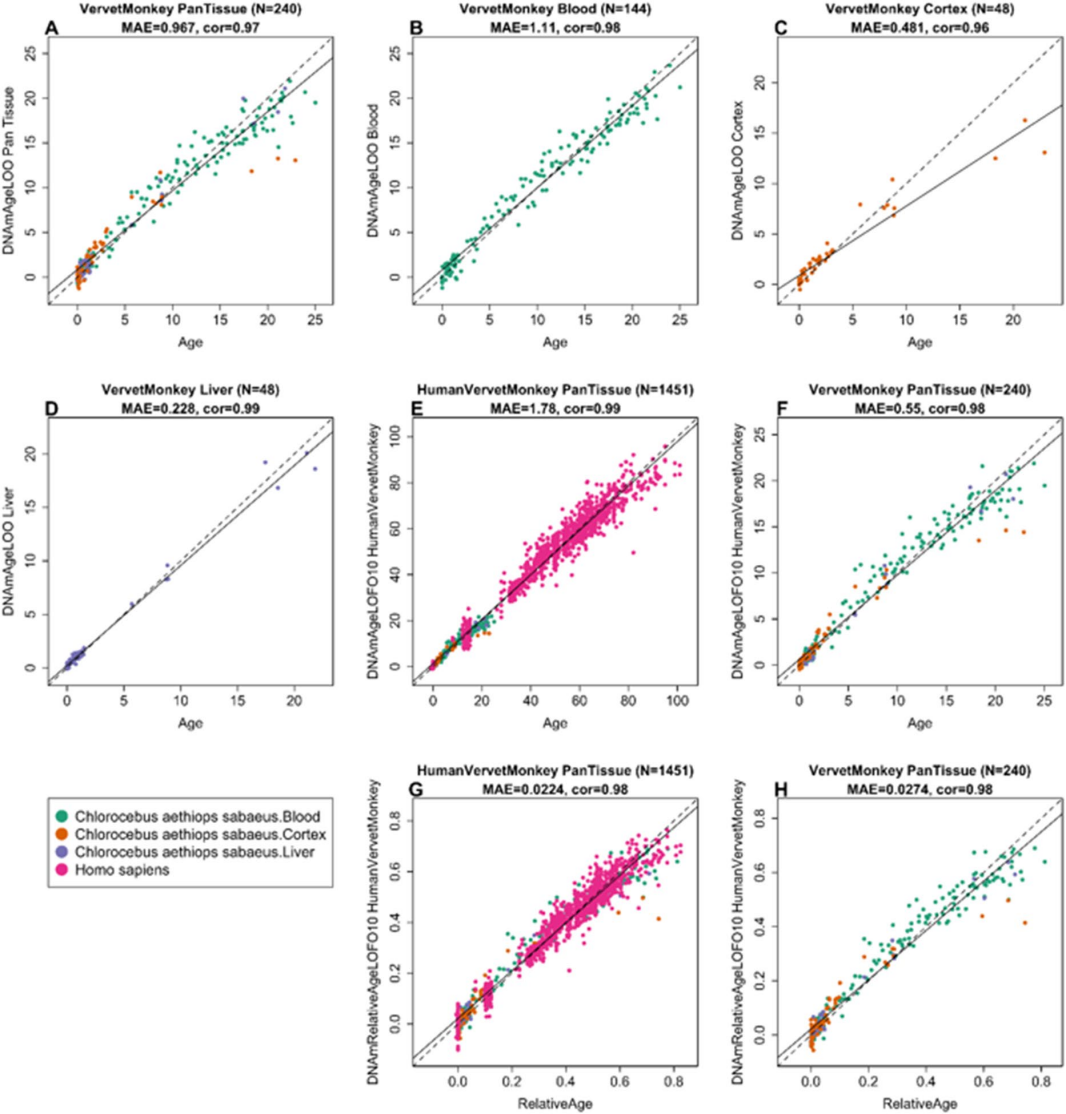
Cross-validation study of epigenetic clocks for vervet monkeys and humans. **A**–**D** Four epigenetic clocks that only apply to vervet. Leave-one-sample-out estimate of DNA methylation age (*y*-axis, in units of years) versus chronological age for **A** all available vervet tissues, **B** vervet blood, **C** vervet cerebral cortex, **D** vervet liver. Tenfold cross-validation analysis of the human-vervet monkey clocks for **E**, **F** chronological age and **G**, **H** relative age, respectively. **E**, **G** Human samples are colored in magenta and vervet samples are colored by vervet tissue type, and analogous in **F**, **H** but restricted to vervet samples (colored by vervet tissue type). Each panel reports the sample size (in parentheses), correlation coefficient, median absolute error (MAE)

**Fig. 2:**
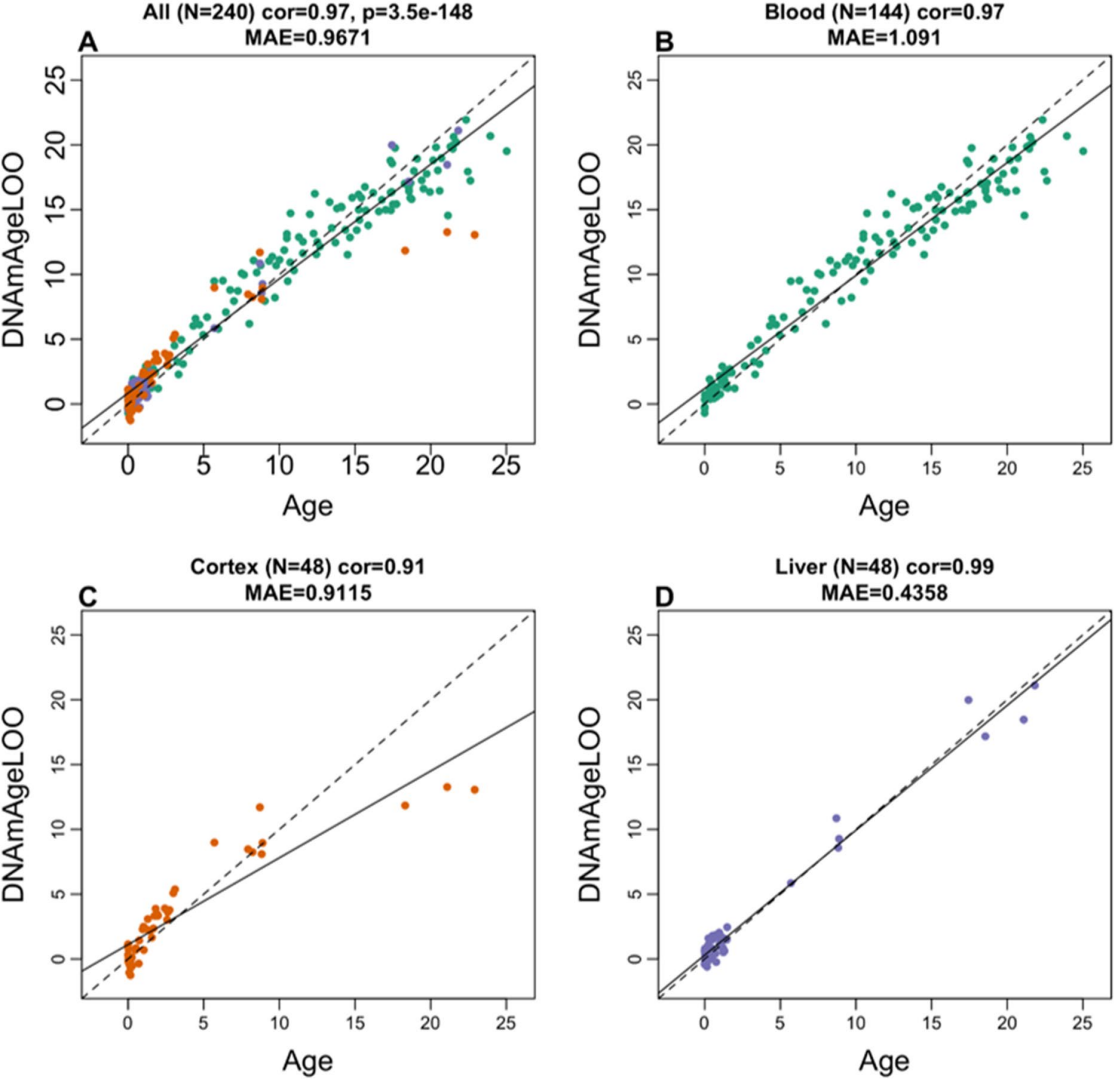
The multi-tissue epigenetic clock for vervets applied to individual tissues. Leave-one-sample-out estimate of age based on DNA methylation data (*x*-axis) versus chronological age (in units of years) for **A** all tissues, **B** blood, **C** cerebral cortex, **D** liver. Each panel reports the sample size, Pearson correlation coefficient, and median absolute deviation (median error)

To arrive at unbiased estimates of the epigenetic clocks, we used leave-one-out (LOO) cross-validation of the training data. The cross-validation study reports unbiased estimates of the age correlation *R* (defined as Pearson correlation between the age estimate (DNAm age) and chronological age) as well as the median absolute error (mae) measuring the deviation between the predicted and observed age (for chronological age in years). As indicated by its name, the vervet multi-tissue clock is highly accurate in age estimation of the different tissue samples (*R* = 0.98 and median error 0.89 years, Fig. 1A). The multi-tissue clock also performs well when restricting the analysis to samples from a given tissue type: *R* = 0.98 in the blood, *R* = 0.99 in the liver, and *R* = 0.91 in the brain cortex (Fig. 2). We also developed highly accurate vervet clocks for single tissues: blood (*R* = 0.98, Fig. 1B), cerebral cortex (*R* = 0.95, Fig. 1C), and liver (*R* = 0.99, Fig. 1D). The accuracy of the multi-tissue clock for the vervet (*r* = 0.98) exceeded the accuracy of the human pan-clock (*r* = 0.96) [13] and the accuracy of mouse multi-tissue clocks, which have been reported to range from *r* = 0.79 to *r* = 0.89 [79].

We developed two dual species clocks based on our vervet samples and previously characterized human tissues [45, 65, 66]: the human-vervet clock for chronological age (*R* = 0.99 for the human and vervet samples and *R* = 0.98 for the vervet samples, Fig. 1E, F) and relative age (*R* = 0.98 for the human and vervet samples and *R* = 0.97 for the vervet samples, Fig. 1G, H).

We tested the performance of the vervet blood clock in longitudinal blood samples from 14 individuals collected at two different time points. In these samples, we correlated the changes in DNAm age predicted on the basis of the vervet blood clock with the changes in the actual chronological age (Supplementary Fig. 2). In all pairs of samples from the same animal, the samples collected later were correctly predicted to be from an older animal.

### Vervet clock applied to other primates

To determine the cross-tissue performance and the cross-species conservation of the vervet multi-tissue clock, we applied the vervet pan-clock to an array of tissues from key organs from two primate species: macaque (*N* = 283 samples from eight tissues) and humans (*N* = 1211 from 16 tissues). The data from rhesus macaque are described in a companion paper [80].

**Fig. 3:**
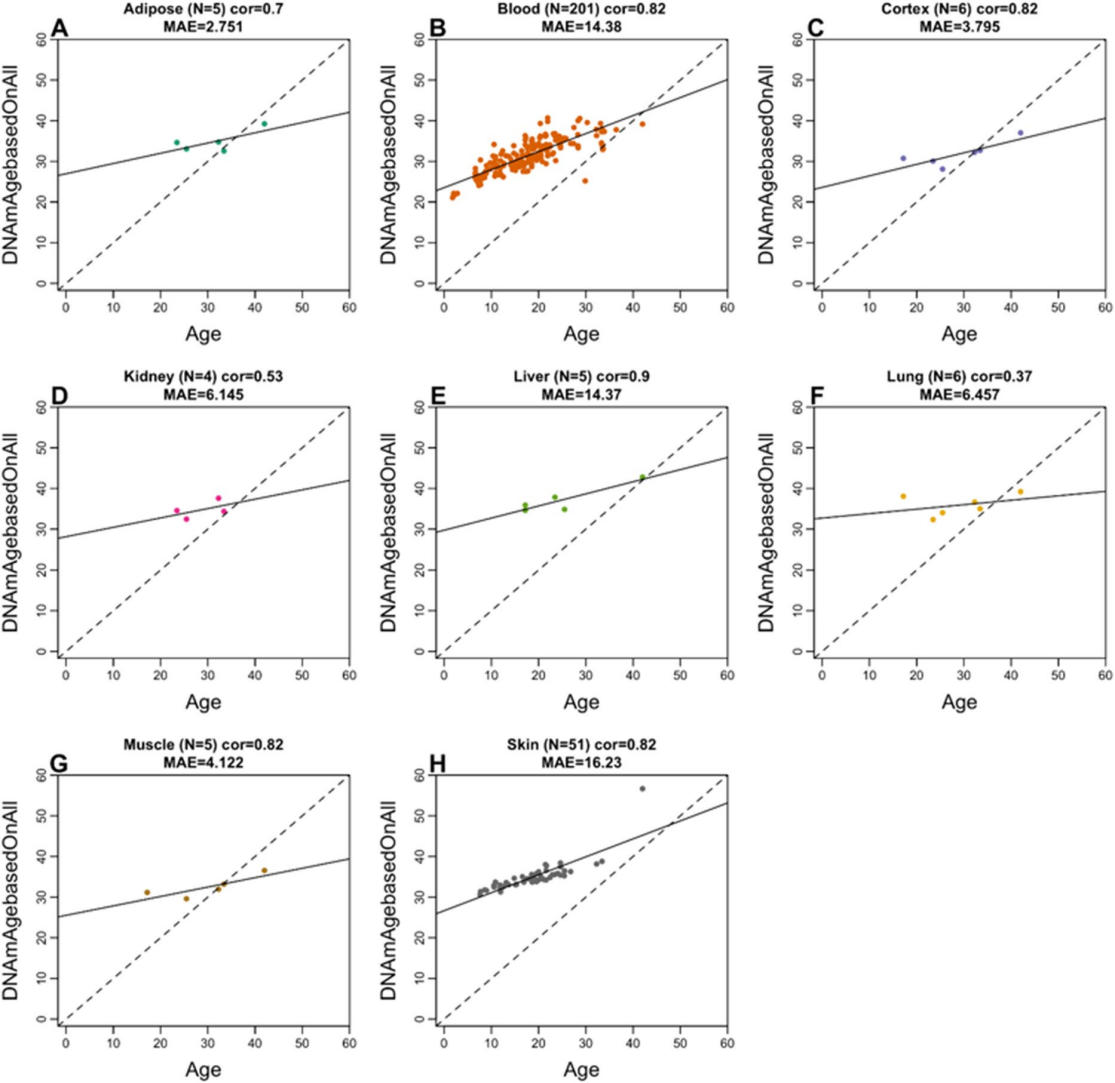
Multi-tissue vervet monkey clock applied to tissues from rhesus macaques. Each dot corresponds to a tissue sample from rhesus macaques: **A** adipose, **B** blood, **C** brain cortex, **D** kidney, **E** liver, **F** lung, **G** muscle, **H** skin. The *y*-axis reports the age estimate according to the multi-tissue vervet clocks. The predicted DNAm age in macaque tissues according to the vervet pan-clock (*y*-axis) and chronological age of the rhesus specimens (*x*-axis). The number of samples is shown in parentheses; cor, Pearson’s correlation; MAE, median absolute error

**Fig. 4:**
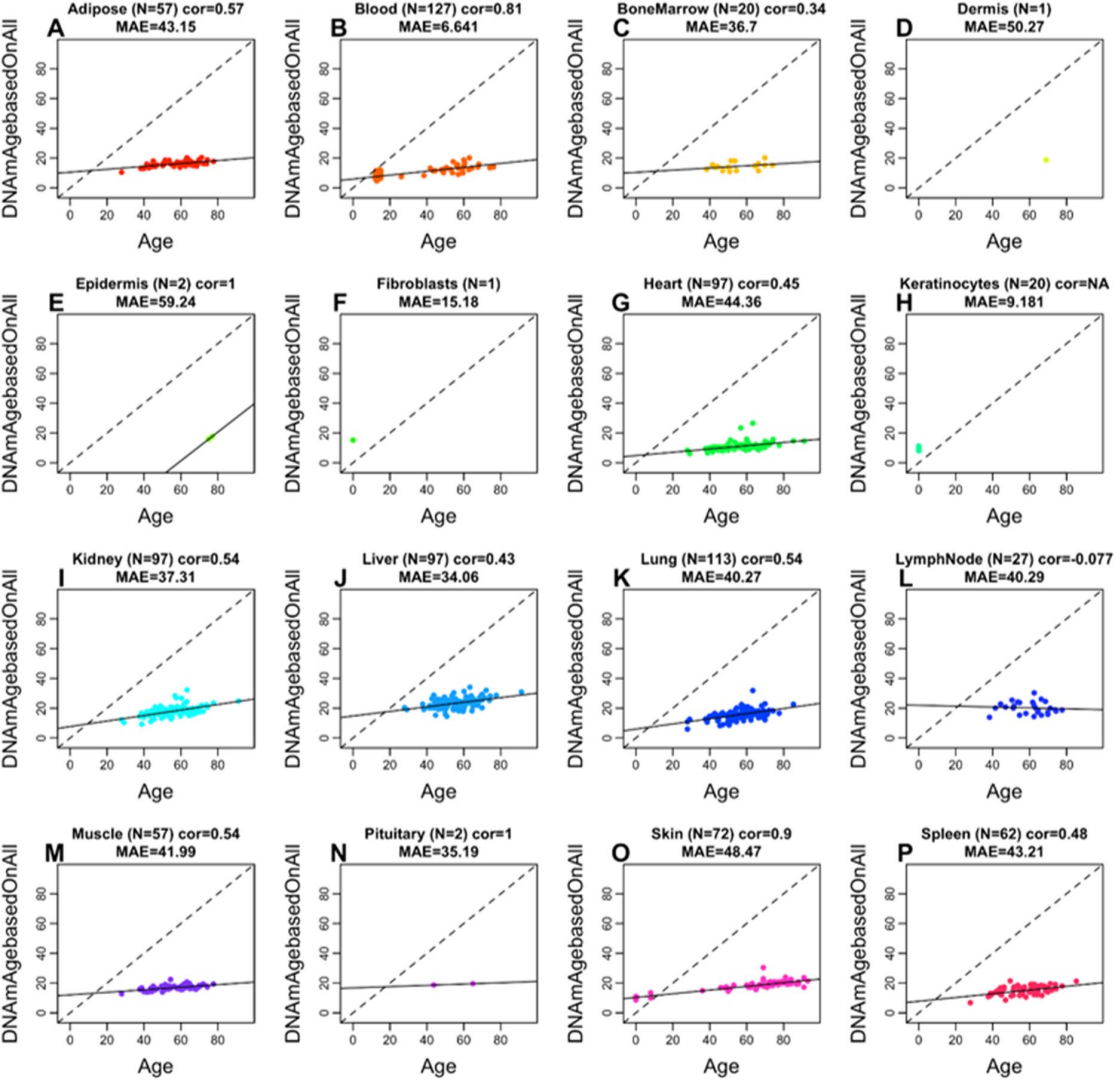
Multi-tissue vervet clock applied to 16 tissue types from humans. Each dot corresponds to a human tissue samples. The predicted DNAm age in human tissues according to the vervet multi-tissue clock (*y*-axis) and chronological age of the human specimens (*x*-axis). The number of samples is shown in parentheses; cor, Pearson’s correlation; MAE, median absolute error

We observed an overall moderate to high correlation between the chronological age and predicted age based on the vervet multi-tissue clock: *R* = 0.76 for macaque (Supplementary Fig. 3) and *R* = 0.64 for human (Supplementary Fig. 4). High correlations in individual tissues could be observed in blood (*R* = 0.82 in macaque and *R* = 0.81 in humans) and in skin (*R* = 0.82 in macaque and *R* = 0.9 in humans) (Fig. 3, Fig. 4). Strictly speaking, it is not possible to compare the correlation coefficients across the different tissues since it depends on the underlying age distribution (e.g., minimum and maximum age) and to a lesser extent on the sample size. The vervet clock is poorly calibrated in other primates such as rhesus macaque and human as reflected by an “offset” that leads to a high median error in many tissues (Fig. 3, Fig. 4). However, the vervet clock leads to moderately high correlation coefficients in rhesus adipose (*R* = 0.7), blood (*R* = 0.82), brain cortex (*R* = 0.82), liver (*R* = 0.9), muscle (*R* = 0.82), and skin (*R* = 0.82) (Fig. 3).

### Age-related CpGs in vervets

**Fig. 5:**
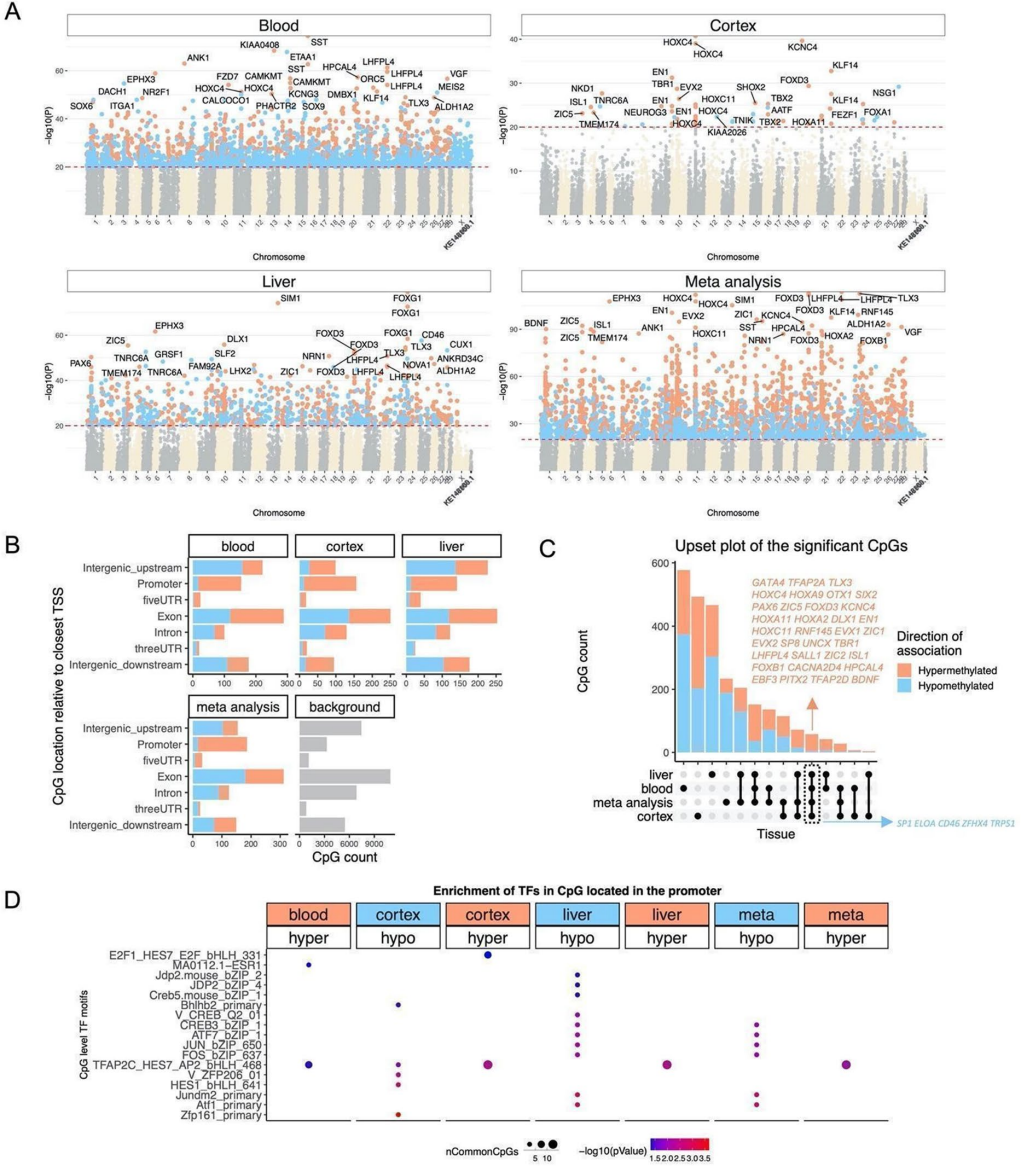
Epigenome-wide association study of age in tissues from *Chlorocebus sabaeus*. **A** Manhattan plots of the EWAS results in different tissues. Stouffer meta-analysis was used to combine the results across different tissues. The coordinates are estimated based on the alignment of Mammalian array probes to ChlSab1.1.100 genome assembly from ENSEMBL. The direction of associations with *p* < 10 × ^−20^ (red dotted line) is colored in red (increased methylation with age) and blue (decreased methylation). The top 30 CpGs were labeled by their neighboring genes. **B** Location of top CpGs in each tissue relative to the closest transcriptional start site. Top CpGs were selected at *p* < 10^−10^ and further filtering based on *z* score of association with chronological age for up to 500 in a positive or negative direction. The number of selected CpGs: blood, 1000; cortex, 777; liver, 1000; meta-analysis, 1,000. The gray color in the last panel represents the location of 35,898 mammalian BeadChip array probes mapped to ChlSab1.1.100 genome. **C** Upset plot representing the overlap of aging-associated CpGs based on meta-analysis or individual tissues. Neighboring genes of the overlapping CpGs were labeled in the figure. **D** Transcriptional motif enrichment for the top CpGs in the promoter and 5′ UTR of the neighboring genes. The motifs were predicted using the MEME motif discovery algorithm, and the enrichment was tested using a hypergeometric test [72]. In total, 19,087 CpGs were predicted to be located on the motifs and were used as the background. nCommonCpGs indicates the number of target CpGs that overlapped with the background CpGs on the analyzed motif

In total, 36,727 probes from HorvathMammalMethylChip40 were aligned to specific loci proximate to 6,110 genes in the vervet monkey (*Chlorocebus_sabaeus.ChlSab1.1.100*) genome. Epigenome-wide association studies (EWAS) of chronological age revealed that the age-related changes in DNAm are to a marked extent tissue-specific in the vervet monkey (Fig. 5A).

Pairwise scatter plots of the age EWAS signals (Supplementary Fig. 5) revealed a moderate positive correlation among the peripheral tissues, blood, and liver (*r* = 0.63), whereas the correlations among the peripheral tissues and the brain cortex were markedly lower, i.e., liver and cortex (*r* = 0.21) and blood and cortex (*r* = 0.14). However, the moderate to low conservation in our analyzed tissue types may reflect a moderate sample size in non-blood tissues.

The age EWAS results suggest that the cerebral cortex has the lowest number of DNAm changes associated with age (*N* = 916) compared to blood (*N* = 12,334) and liver (*N* = 4,454) at a nominal *p*-value < 10^−10^. This lower number of the age-associated DNAm alterations is probably not due to statistical differences because both brain and liver had the same number of samples (*N* = 48) and with similar age distribution (Table 1). Rather, we hypothesize that it can be attributed to the post-mitotic state of neurons in the brain compared to blood and liver cells. However, the lower number of age-related CpGs in the brain could reflect heterogeneity of cell types in the bulk cortical brain tissues or technical issues [81, 82] that are difficult to dissect with absolute consistency.

The genomic localization of top age-related DNAm changes and the proximate genes in each tissue are as follows (Fig. 5A). The most significant EWAS signals in the blood are in a *KIAA0408* exon (*z* = 19.1) and in a promoter of the *SST* gene (*z* = 18.3), which is encoding somatostatin acting as a negative regulator of growth hormone slowing aging in humans [83]. In the cerebral cortex, the strongest EWAS signals are in an exon of the *KCNC4* gene (*z* = 13.3) and in a promoter of the *HOXC4* gene (*z* = 13.5), which is a homeobox gene crucial during erythroid lineage differentiation [84] and which expression is associated with age in bone marrow stromal cells [85] and skin cell differentiation and tumors [86]. Given the identification of a gene involved in erythroid lineage among the brain clock sites, it is pertinent to note that the brains were perfused to remove blood prior to dissection. The top EWAS signals in the liver are in a promoter of the *FOXG1* gene (*z* = 18.9), which mutations cause a severe neurodevelopmental disorder, the Rett syndrome [87], and in an intron of the *SIM1* gene (*z* = 18.3).

In the meta-analysis across these three tissue types, the top age-related DNAm changes included gain of methylation in *LHFPL4* exon (*z* = 22.7), an exon of the *FOXD3* gene (*z* = 22.6), which is a transcription repressor essential in embryogenesis controlling the multipotent mammalian neural crest, neuronal differentiation, and fate [88–90], and a promoter of the *TLX3* gene (*z* = 22.6), which is a transcription factor acting as a master regulator of neuronal differentiation in embryonic development and in embryonic stem cells [91, 92].

GREAT enrichment analysis revealed that CpGs that gain methylation with age across several tissues are located near targets of the Polycomb proteins SUZ12 (BENPORATH_SUZ12_TARGETS) and EED (BENPORATH_EED_TARGETS) and genes possessing the trimethylated H3K27 mark in their promoters (BENPORATH_ES_WITH_H3K27ME3, Supplementary Fig. 6). Polycomb repressive complexes are involved in chromatin remodeling resulting in epigenetic silencing of genes, such as homeobox genes, and have been implicated in the modulation of brain aging [93]. Highly significant enrichment could also be observed for genes involved in DNA binding (sequence-specific DNA binding, transcription factor activity), nervous system (nervous system phenotype, abnormal nervous system morphology), and development (lethality during fetal growth through weaning, Supplementary Fig. 6).

We analyzed the distribution of age-associated CpGs in different tissues across different genomic regions, including promoters, UTRs, exons, introns, and intergenic sequences (Fig. 5B). The most significant age-related DNAm changes were gain of methylation in the promoters and 5′ UTRs, which is consistent with what has been observed in other species [94].

Using a generalization of Venn diagrams (upset plot analysis), we identified CpGs that showed consistent age-associated DNAm changes in multiple tissues (Fig. 5C). We observed 58 of such shared CpGs undergoing age-related DNAm alterations in the blood, cerebral cortex, and liver which were located next to 39 genes of which 34 and 5 exhibited a gain and loss of methylation, respectively.

The top age-associated CpGs included gain of methylation in *LHPL4* exon, *FOXD3* exon, and *TLX3* promoter and loss of methylation in *SP1* exon, a CpG downstream of *CD46*, and *TRPS1* intron (Fig. 5C). Some of these genes are implicated in aging phenotypes. For example, *SP1* is a key regulator of mTORC1/P70S6K/S6 signaling pathway [95, 96] and is involved in several aging-associated diseases including cancer [97], hypertension [98], atherosclerosis [99], Alzheimer’s [100], and Huntington diseases [101].

We examined the transcriptional factor motifs enriched for the top CpGs located in promoters or 5′ UTRs with DNAm changes in either direction of each tissue (Fig. 5D). The top TF motif most significantly enriched for the top EWAS CpGs was Zfp161 (ZBTB14 in human) motif loss of methylation with age in the cerebral cortex. Further, loss of methylation CpGs showed a strong enrichment for Atf1 and several immune-related TF motifs such as Jundm2, FOS, JUN, and CREB in the liver. TFAP2C was a TF motif associated with loss of methylation in all tissues. This motif is involved in cell-cycle arrest and germ cell development, and it is implicated in several types of cancer [102, 103].

## Discussion

Leveraging the mammalian methylation array (HorvathMammalMethylChip40) [67], we generated DNA methylation data from three tissue types (brain, blood, liver) in the vervet monkey. These samples represent the most comprehensive dataset thus far of methylomes in vervets across multiple tissues and ages. We obtained high-quality DNAm data, as reflected in the perfect clustering pattern of the samples by tissue type without any intermixture between different tissue samples within the clusters.

Using these DNAm data, we trained and validated highly accurate age estimators (epigenetic clocks) that apply to the entire life course (from birth to old age) and identified genes associated with the aging process in the vervet. These data allowed us to construct a highly accurate multi-tissue age estimator (pan-clock) based on three vervet tissue types (brain, blood, liver) and clocks developed based on individual vervet tissues. Given that the vervet pan-clock can estimate age in 3 different tissues, we anticipate that it applies to additional tissues as well. However, we cannot rule out that these clocks could fail in some highly specialized cell types. Epigenetic age estimators that focus on specific tissues or cell types can have greater accuracy than multi-tissue age estimators [104].

We found evidence that epigenetic aging is slowed in cerebral cortex compared to blood and liver, as can be seen from comparing the dashed line (*y* = *x*) with the solid line (linear regression line) in Fig. 1A–D. A similar phenomenon has been observed in humans where brain tissue (especially the cerebellum) ages more slowly than other parts of the body [104, 105]. Our EWAS analysis provided a CpG level analysis of aging effects (Fig. 5). Compared to liver and blood, the vervet cortex exhibited fewer negatively age-related CpGs but more positively age-related CpGs (Fig. 5C). The EWAS of age in vervets shows that chromosome regions targeted by Polycomb repressive complex 2 gain methylation with age. This enrichment has also been observed for prior human-specific clocks such as the pan tissue clock [13].

The vervet pan-clock showed a positive correlation of age estimates in a wide range of tissues in macaque and humans (except the lymph nodes in humans), despite the phylogenetic distance of ~ 12 million years between the vervet and macaque and ~ 29 million years between the vervet and human lineages [106]. Figures 3 and 4 show that when analyzed using the vervet pan-tissue CpG set, positive correlations between epigenetic age and chronological age can be observed in both macaques and humans but the slopes (rate of change) are low and the MAE values are unacceptably high. For rhesus tissues, we recommend to use rhesus clocks that have been published elsewhere [28].

These epigenetic clocks reveal several salient features with regard to the biology of aging. First, the vervet multi-tissue clock re-affirms the implication of the human multi-tissue clock, which is that aging might be a coordinated biological process that is harmonized throughout the body. Second, the ability to combine these two multi-tissue clocks into a single human-vervet multi-tissue clock attests to the high conservation of the aging process across two evolutionary distant primate species, whose lineages diverged ~ 29 million years ago [106].

Epigenetic clocks for humans have found many biomedical applications including the measure of age in human clinical trials [15, 107]. This instigated development of similar clocks for mammals such as mice [79, 108–112]. While rodent models have obvious advantages, it can be challenging to translate findings from rodents to primates [113, 114]. NHPs play an indispensable role in aging studies and preclinical work of anti-aging treatments [115, 116]. Lifespan and healthspan studies, as well as assessments of anti-aging interventions in primates, remain costly and time consuming. The development of suitable biomarkers promises to greatly reduce the costs and time needed for carrying out studies in these primates. To increase the chance that findings in vervets translate to humans, we created dual species clocks, human-vervet clocks, for absolute and relative age. The bias due to differences in maximum lifespan is mitigated by the generation of the human-vervet clocks for *relative* age clock, which embeds the estimated age in context of the maximal lifespan recorded for the relevant species. The high accuracy of these clocks demonstrates that one can build epigenetic clocks for two species based on a single mathematical formula. Treatments that alter the epigenetic age of vervets according to our human-vervet clocks are likely to exert similar effects in humans.

Beyond the laboratory, vervet monkeys from wild populations are increasingly used in biomedical and anthropological research [117]. Tooth eruption patterns are typically used as a practical predictor of developmental stage in wild vervets and other NHPs [118, 119]. Although these patterns approximate the developmental stage of an individual, their utility is limited in terms of accurate prediction of chronological age, particularly in adults with a fully developed dental pattern, in which distinguishing among various stages of adulthood and senescence is difficult. In animals of unknown chronological age, the epigenetic clock can enable more accurate estimates of chronological age and thus decrease the confounding effects of age, increase the statistical power of analysis, and decrease the number of animals needed for studies, according to the “three Rs”: replacement, reduction, and refinement. In addition, it can improve monitoring of health status in natural populations, provide insight into life history, and enable identification of lifespan modulating factors in the context of a natural habitat. The genetic architecture of VRC vervets, whose tissues were used for the clock construction, is simplified compared to that of wild vervets due to genetic bottlenecks [47]. While the methods used to create the clock presumably favors methylation sites for which genetic variance is low, one concern is that the bottlenecks may have driven certain genetic variants to fixation in the VRC. We are presenting a prototypical application of the various vervet clocks to wild vervets. However, we caution the reader that epigenetic age estimates may be affected by technical issues and biological differences (genetic differences, diet, exposure to pathogens, other environmental factors) that typically lead to an “offset,” i.e., a constant difference between epigenetic age estimate and the true chronological age. Future studies should evaluate the effect of genetic background and other potential confounders in wild vervet populations.

We expect that the availability of these clocks will provide a significant boost to the attractiveness of the vervet as a translational model for health, developmental, and aging research. The vervet pan-clock and tissue-specific clocks are biomarkers that can facilitate studies of the course of biological aging in the context of various genetic factors and environmental exposures (for example, preclinical testing of rejuvenating therapies).

## Supplementary Information

Below is the link to the electronic supplementary material.

Supplementary file1 (DOCX 809 kb)

## Data Availability

The data will be made publicly available on Gene Expression Omnibus as part of the data release from the Mammalian Methylation Consortium. Genome annotations of these CpGs can be found on Github https://github.com/shorvath/MammalianMethylationConsortium. The mammalian methylation array is broadly available to the research community from the non-profit Epigenetic Clock Development Foundation (https://clockfoundation.org/).
