# Supplementary material for: Epigenetic clock and methylation studies in vervet monkeys
Source: GeroScience. 2021 Sep 30;44(2):699–717. doi: 10.1007/s11357-021-00466-3 (PMC9135907; doi:10.1007/s11357-021-00466-3)
Supplement: Supplementary file 1 — Supplementary file1 (DOCX 809 kb) [file 11357_2021_466_MOESM1_ESM.docx]

**SUPPLEMENTARY MATERIAL**

**for "Epigenetic clock and methylation studies in vervet monkeys"**

**
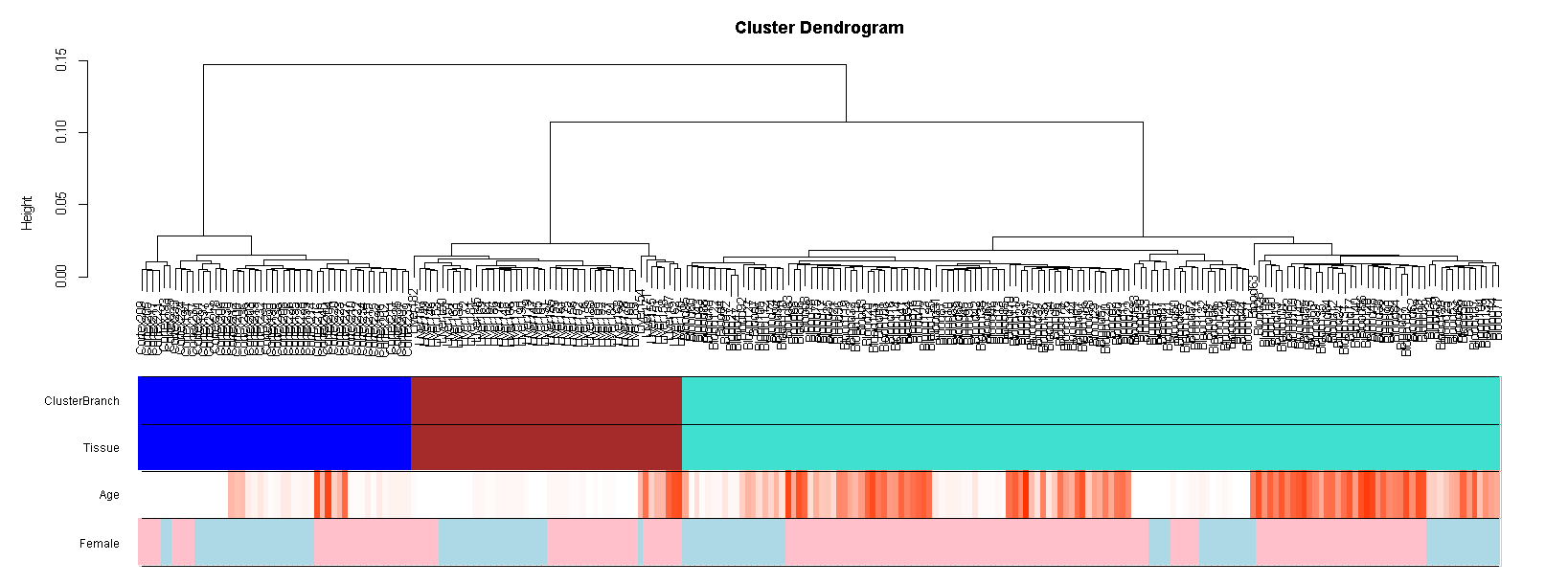
**

**Supplementary Figure 1**. Unsupervised hierarchical clustering of tissue samples. Average linkage hierarchical clustering based on the interarray correlation coefficient (Pearson correlation). A height cut-off of 0.05 led to branch colors that correspond to Tissue type (second color band: blue - brain cortex from day 0 to 22 years of age, maroon – liver from day 0 to 21 years of age, turquoise – blood from day 1 to 25 years of age), sex (females – pink, males – blue). Age is shown in the scale from youngest (white) to oldest (red).


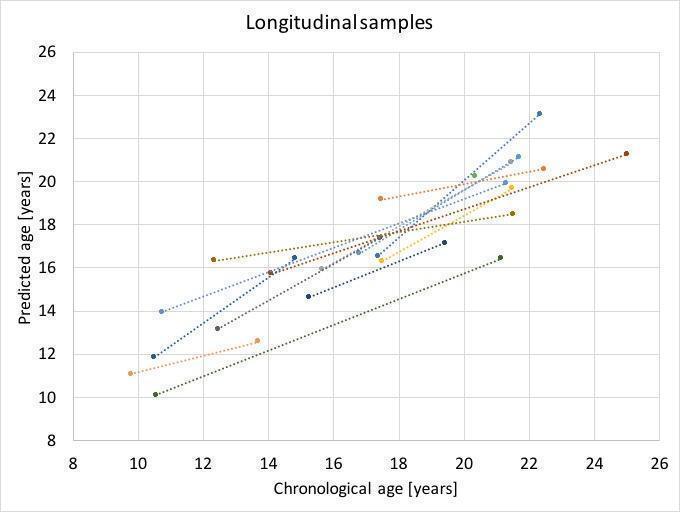


**Supplementary Figure 2.** The DNAm age predictions by using the blood-specific clock in pairs of blood samples collected from the same animals at two different time points. Predicted age (y-axis) is shown relative to chronological age (x-axis) for pairs of samples collected from 14 individuals.


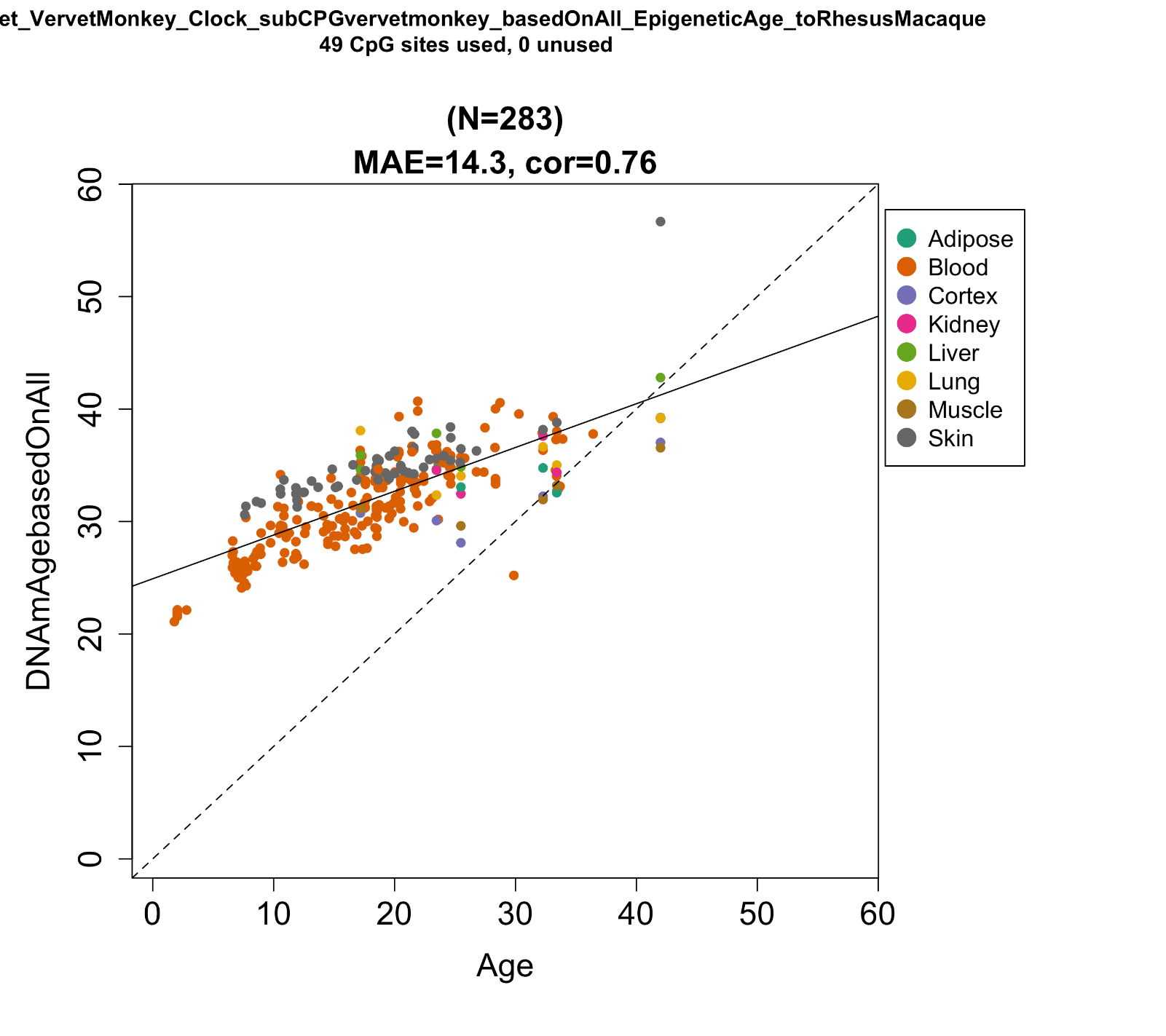


**Supplementary Figure 3.** **Vervet pan-clock applied to macaque tissues**. The predicted DNAm age in human tissues according to the vervet pan-clock (y-axis) and chronological age of the human specimens (x-axis). The number of samples is shown in parentheses; cor – Pearson’s correlation, MAE – median absolute error.


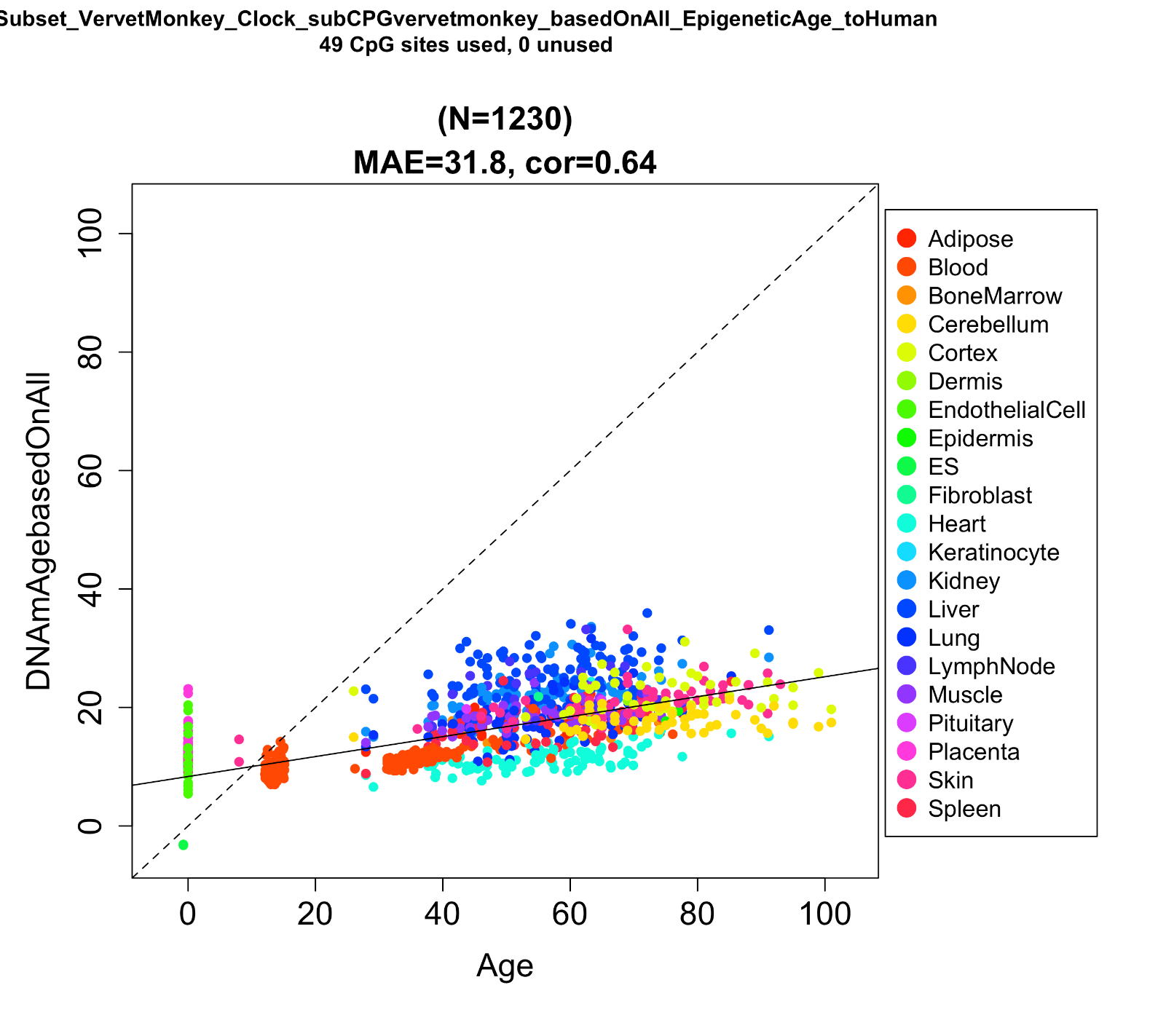


**Supplementary Figure 4.** The conservation DNAm age estimated with the vervet pan-clock in human tissues. The predicted DNAm age in human tissues according to the vervet pan-clock (y-axis) and chronological age of the human specimens (x-axis). The number of samples is shown in parentheses; cor – Pearson’s correlation, MAE – median absolute error. Panels correpond to different human tissue types.


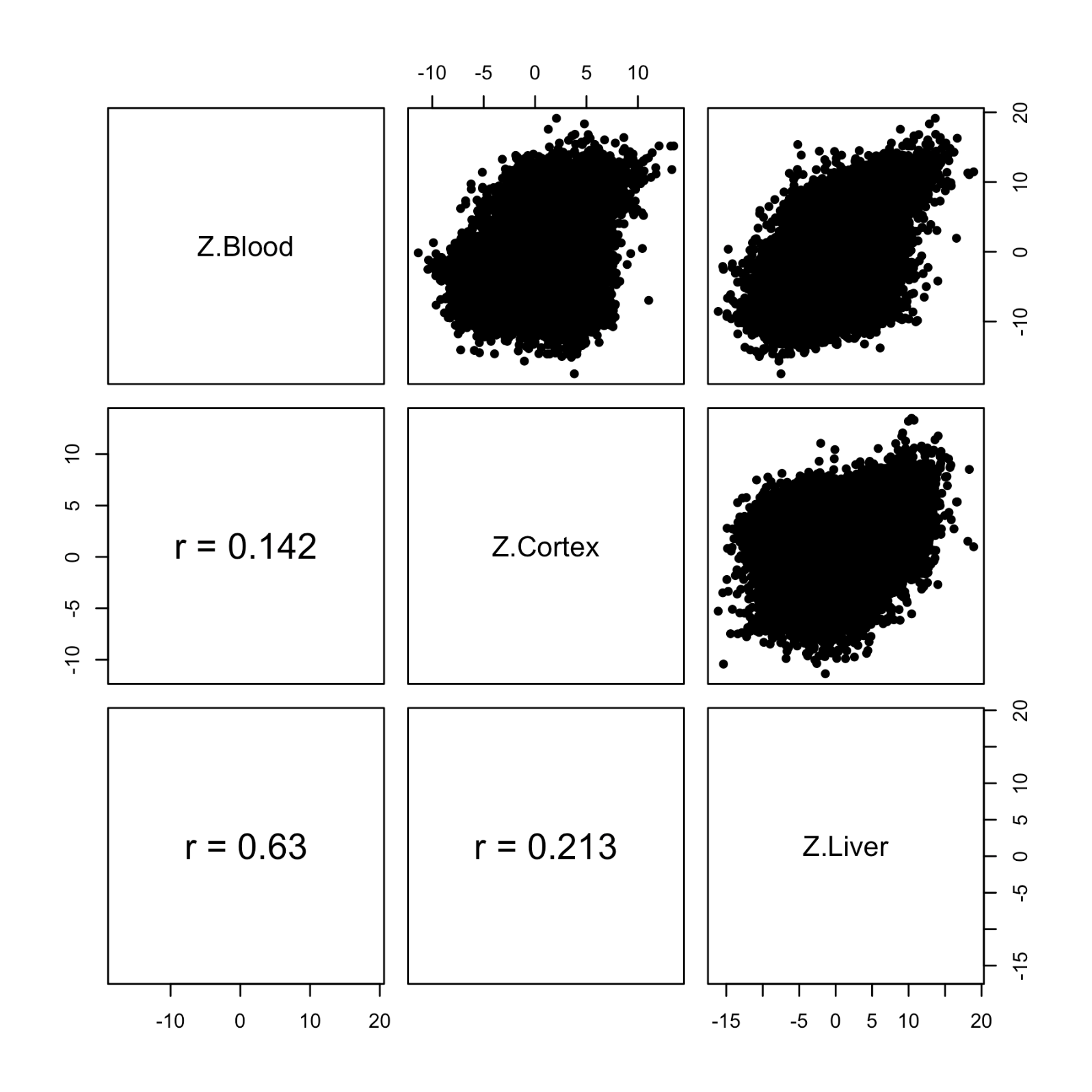


**Supplementary Figure 5. Epigenome wide association study of correlation in three different tissues**. Each dot corresponds to a CpG. Z statistics for a correlation test of age in the blood, cerebral cortex, and liver. Pairwise scatter plots reveal a strong positive correlation (r=0.63) between EWAS results in the blood and brain tissue.

**
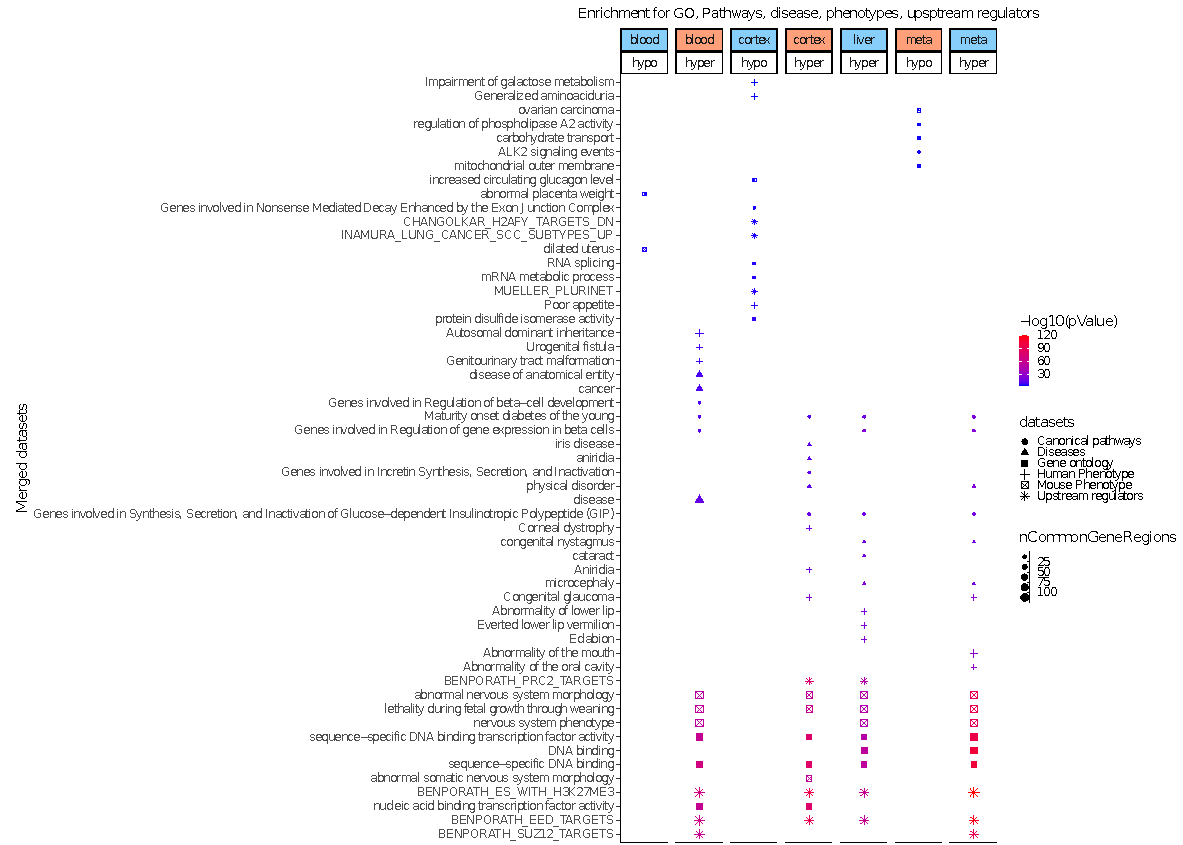
**

**Supplementary Figure 6. Enrichment analysis of the top CpGs associated with DNAm age in each tissue and in meta analysis.** The analysis was done using the genomic region of enrichment annotation tool [(McLean et al. 2010)](https://paperpile.com/c/UwtSqM/Y7EWB). The gene level enrichment was done using GREAT analysis [(McLean et al. 2010)](https://paperpile.com/c/UwtSqM/Y7EWB) and human Hg19 background. The background probes were limited to 24,799 probes that were mapped to the same gene in the vervet monkey genome. The top three enriched datasets from each category (Canonical pathways, diseases, gene ontology, human and mouse phenotypes, and upstream regulators) were selected and further filtered for significance at p < 10^-4^.

**Technical Details surrounding the DNAm age estimator**

**Statistical methods used for building the clocks**

The epigenetic clocks were used by employing a single elastic net regression model analysis (R function glmnet). We use used Leave-one-out analysis (LOO) using a single lambda value. We chose the following parameters for the glmnet R function (Alpha: 0.5, CV Fold: 10, Lambda choice for Clock: 1 standard error above minimum CV-MSE).

**Covariates and coefficient values of the vervet clocks**

1. The vervet pan tissue clock is based on 48 CpGs whose coefficient values are specified in the column "Coef.VervetPanTissue". Age transformation=identity, i.e. F(Age)=Age
2. Tissue specific clocks for vervet have been created for liver (83 CpGs), blood (97 CpGs), cerebral cortex (33 CpGs).
3. The human vervet clock for chronological age is based on 570 CpGs whose coefficient values are specified in the column "Coef.HumanVervetLogLinearAge". Age transformation=log-linear described below.
4. The final human vervet clock for relative age is based on 519 CpGs whose coefficient values are specified in the column "Coef.HumanVervetBloodRelativeAge". Age transformation: relative age. i.e. F(Age)=Age/maxLifespan. Max lifespan for vervets is 30.8 years. Human max lifespan =122.5 years.

**General description of age transformation**

The human-vervet clocks for chronological age used log linear transformations that are similar to those employed for the HUMAN pan tissue (Horvath 2013) [2].

An elastic net regression model (implemented in the glmnet R function) was used to regress a transformed version of age on the beta values in the training data. The glmnet function requires the user to specify two parameters (alpha and beta). Since I used an elastic net predictor, alpha was set to 0.5. But the lambda value of was chosen by applying a 10 fold cross validation to the training data (via the R function cv.glmnet).

The elastic net regression results in a linear regression model whose coefficients b_0_, b_1_, . . . , relate to transformed age as follows
*F*(chronological age)=*b*_0_*+b*_1_*CpG*_1_*+ . . . +b*_p_*CpG*_p_+error

Note that the intercept term is denoted by b_0_. The coefficient values can be found in the attached Excel file.

Based, on the coefficient values from the regression model, DNAmAge is estimated as follows
*DNAm*Age=$F^{-1}$(*b*_0_*+b*_1_*CpG*_1_*+ . . . +b*_p_*CpG*_p_)

where $F^{-1}\left( y \right)$ denotes the mathematical inverse of the function F(.). Thus, the regression model can be used to predict to transformed age value by simply plugging the beta values of the selected CpGs into the formula.

### **Defining Properties of the log linear transformation**

As indicated by its name, the “log-linear” function, has a logarithmic dependence on age before the average age of sexual maturity (of the species) and a linear dependence after Age at Sexual Maturity (of the species). For the human-vervet clocks we used the following averages at sexual maturity (in units of years): 13.5 years for humans and 3.916 years for vervets.

### Construction

We used a piecewise transformation, parameterized by Age of Sexual Maturity ($A$).

The transformation is F(x), given by

$$F\left( x \right)=g\left( \frac{x+1.5}{A+1.5} \right)\text{ where }g\left( t \right)= \left\{ \begin{aligned} \begin{aligned} \begin{aligned} \log\left( t \right), for 0\leq t\leq1 \\ t-1, for 1\leq t \end{aligned} \end{aligned} \end{aligned} \right.$$

Explicitly, F(x) is given by

$$F\left( x \right)=\left\{ \begin{aligned} \begin{aligned} \begin{aligned} \log\left( \frac{x+1.5}{A+1.5} \right), for 0\leq x\leq A \\ \frac{x-A}{A+1.5}, for A\leq x \end{aligned} \end{aligned} \end{aligned} \right.$$

In order to use this transformation to predict Age on *new samples*, one needs to use the *inverse* transformation, F^-1^(y), given by

$$F^{-1}\left( y \right)= \left\{ \begin{aligned} \begin{aligned} \begin{aligned} \left( A+1.5 \right)*\text{exp}\left( y \right)-1.5, for y\leq0 \\ (A+1.5)y+A, for y\geq0 \end{aligned} \end{aligned} \end{aligned} \right.$$

For predicting age, apply the inverse transformation to coefficient-weighted sum. That is,

$$DNAmAge=F^{-1}\left( x*\beta\right)$$

where $\beta$ is the vector of coefficients and $x$ is the vector of methylation values, with an intercept term.

## The DNAm Age estimate is estimated in two steps.

First, one forms a weighted linear combination of the CpGs whose details can be found in Table

The table reports the probe identifier (cg number) used in the custom Infinium array (HorvathMammalMethylChip40). The weights used in this linear combination are specified in the respective column entitled "Coef.".

The formula assumes that the DNA methylation data measure "beta" values but the formula could be adapted to other ways of generating DNA methylation data.
